# Effects of non-thermal plasma on disinfection of indoor air and reduction of particulate matter

**DOI:** 10.1038/s41598-026-52317-w

**Published:** 2026-05-10

**Authors:** Wei Liu, Mengmeng Wang, Zhongyi Xie, Ye Lu, Guoqing Hu, Junming Lin, Xieshang Pan, Ji Xu, Ye Li

**Affiliations:** 1 Department of Environmental Health, Zhejiang Provincial Center for Disease Control and Prevention, Hangzhou, 310051 China; 2 Department of Infection Control, The Second Affiliated Hospital of Zhejiang University School of Medicine, Hangzhou, 310051 China

**Keywords:** Non-thermal plasma (NTP), Indoor air, Bacteria, Particulate matter (PM), Disinfection, Diseases, Environmental sciences, Microbiology

## Abstract

Indoor airborne microorganisms and particulate matter (PM) are associated with respiratory infections and non-communicable diseases, posing a significant public health concern. This study evaluated the effectiveness of non-thermal plasma (NTP) for air disinfection and PM removal under real-world conditions. Chamber experiments were conducted using air artificially contaminated with PM, and reduction rates were compared between NTP and control groups. Field investigations were performed in an unoccupied classroom and a clinic during normal operation, and airborne bacterial concentrations, PM levels, and influence of human activities and environmental factors were evaluated. The results showed that a 30-min NTP treatment reduced approximately 90% of PM_2.5_ concentration in the chamber, after adjustment for natural decay. Under unoccupied conditions, airborne bacterial concentrations were significantly decreased after 90 min of NTP disinfection, when compared with no treatment. Under occupied conditions, although human activities increased bacterial and PM levels, both indicators declined with prolonged disinfection duration. The highest bacterial load occurred in the 1.1–2.1-µm particle-size fraction. Thus, controlling the number of occupants and minimizing unnecessary door opening during clinical procedures may enhance disinfection efficiency. Overall, NTP effectively inactivated airborne bacteria and removed PM, showing promising potential for improving indoor air quality and reducing airborne disease transmission risks.

## Introduction

Airborne transmission of pathogens poses a substantial public health risk, as demonstrated by influenza, Legionnaires’ disease, and coronavirus disease 2019^1–3^. Certain airborne microorganisms can remain viable for hours or even days, enabling long-distance transmission and clustered infections^4,5^. Besides, indoor air particulate matter (PM) is a well-recognized health hazard, and has been associated with frailty, dementia, cardiometabolic multimorbidity, and lung cancer^6–9^. To mitigate the risks posed by poor indoor air quality, public environments have become a major research focus. Numerous studies have characterized the concentration, properties, and microbial composition of airborne particles in hospitals, schools, offices, and poultry facilities^7,10–13^. Air disinfection is an effective method to control indoor bioaerosols. Conventional physical and chemical disinfection methods can inactivate airborne microorganisms, but their application is often limited by environmental and personnel constraints. Natural ventilation by opening windows may be restricted by architectural design. Ultraviolet (UV) irradiation and chemical disinfectants may be harmful to humans and are therefore generally used in unoccupied spaces, mainly after outbreaks occur. Upper-room UV systems avoid direct exposure of occupants to UV irradiation and can be applied in occupied environments; however, their fixed installation is primarily suitable for specific settings such as clinic rooms^14^. Physical disinfection methods (e.g., filtration and adsorption) retain rather than inactivate microorganisms.

In recent years, plasma has emerged as a promising air disinfection method. As the fourth state of matter, plasma is characterized by broad-spectrum antimicrobial activity, high efficiency, and environmental compatibility^15^. Laboratory investigations have demonstrated that non-thermal plasma (NTP) can effectively inactivate airborne *Staphylococcus epidermidis*, SARS-CoV‐2 RNA, *Escherichia coli*,* Mycobacterium smegmatis*, and bacteriophages^16–18^, and can also efficiently eliminate PM and gaseous pollutants such as formaldehyde^19,20^. A notable advantage of NTP is its relative safety for humans and environmental friendliness, making it suitable for the prevention of airborne diseases in public areas^21–23^. Although ozone is a major byproduct generated during NTP operation, it can be effectively removed by using appropriate technical approaches^24^. However, field investigations of NTP for air disinfection remain limited^25,26^. In real-world settings, bioaerosols are more complex than those under laboratory conditions. Temperature and relative humidity are variable, and continuous human activities can deteriorate air quality, thereby affecting the disinfection efficacy of NTP. Nevertheless, the performance of NTP in environments with human activities, as well as its disinfection and purification efficacy in real-world settings require further validation. This study investigated the disinfection and purification performance of NTP in both controlled experiments and real-world settings, exploring the impacts of environmental factors and human activities, and provides evidence for practical application of NTP.

## Materials and methods

### NTP disinfector

An NTP air disinfector equipped with dielectric barrier discharge reactors (380 × 365 × 688 mm, G-XY-HS02, CHAOSANTAI Corporation, China) was used. Indoor air entered the disinfector via the inlet, passed through the plasma reaction zone, and was recirculated back into the room from the outlet. The recirculating airflow rate was 450 m^3^/h, Ozone (O_3_) absorption filters were integrated to limit O_3_ release into the environment. Additional details about the NTP disinfector are presented in our previous study^22^. PM removal experiments and field disinfection tests (with and without occupants) were conducted between September 2024 and July 2025.

### PM removal experiment

PM removal efficiency was evaluated using a chamber test conducted in a 30-m^3^ sealed glass chamber. The chamber was equipped with a ceiling-mounted fan, HEPA filter at the air outlet, particle sampler (8530, TSI, America) positioned centrally at 1.2 m above the floor, and NTP disinfector. PM was generated using a smoke pen (Bright AB Swaddler; Brighter AB, Sweden) and uniformly distributed throughout the chamber with the fan, and the PM_2.5_ (8530, TSI, Inc., USA) concentrations were measured for 1 min at a flow rate of 3.0 L/min.

In the NTP group, samples were collected before NTP disinfector operation (0 min) and at 5-min intervals for 50 min after NTP operation (5–50 min). The control group followed the same sampling schedule without NTP operation. Each experiment was repeated three times. The chamber temperature was 12.1–15.0 °C and relative humidity was 40.6%–46.9%. Natural decay was corrected as follows:

$$E_{t} = W_{t}*(1-η_{t}), η_{t}=\frac{\mathrm{N}c-\mathrm{N}t}{\mathrm{N}c}$$

where E_t_ is the corrected concentration (µg/m^3^) at time t in the NTP group, W_t_ is the measured concentration at time t in the NTP group (µg/m^3^), η_t_ is the natural decay rate at time t in the NTP group, Nc is the initial concentration in the control group (µg/m^3^), and N_t_ is the concentration at time t in the control group (µg/m^3^).

### Field test without occupants

The inactivation efficacy of NTP against bacterial and bacteriophage aerosols had been evaluated in a previous study^22^; the present study mainly assessed the air disinfection efficacy of NTP through field experiments. The test was conducted in a classroom (about 72 m^2^) at the Zhejiang Provincial Center for Disease Control and Prevention, China. After all the occupants (about 20 individuals) had vacated the room (at about 17:20 h), two NTP disinfectors were operated considering the size of the room. Planktonic bacterial concentrations were ascertained using three six-stage Andersen microbial air sampler (TYK-6 H, Yancheng Tianyue Instrumentation Co., Ltd., China). The cut-off diameters for each stage were > 7.0, 7.0–4.7, 4.7–3.3, 3.3–2.1, 2.1–1.1, and 1.1–0.65 μm, respectively.

Samplers were placed diagonally (near the window, center of the room, and near the door) at about 1.2 m height. The sampling duration was 5 min with an airflow of 28.3 L/min. The NTP group was sampled at 0, 30, 60, and 90 min of continuous NTP operation. In the control group, sampling was also initiated after personnel had vacated the room, without NTP disinfection. Planktonic bacteria were collected at the same time points and locations as in the NTP treatment group. The samplers were disinfected with 70% ethanol between each use and sterilized at the end of each day. After sampling, the nutrient agar plates (Qingdao Hope BioTechnology Co., Ltd.) were incubated at 37 °C for 48 h. The experiments were repeated twice on four different days, and temperature and relative humidity (Dwyer 485B, Dwyer Instruments, Inc., Michigan City, IN, USA) were recorded throughout the experiments.

### Field test with occupants

The field test with occupants was conducted in a Pulmonary Function Clinic (about 20 m^2^) at the Second Affiliated Hospital of Zhejiang University School of Medicine, China. One NTP disinfector was operated during routine clinical activity (starting at about 08:15 h). During the test, the pre-installed air disinfection devices in the rooms were switched off, and the NTP air disinfector served as the sole disinfection measure.

**Fig. 1:**
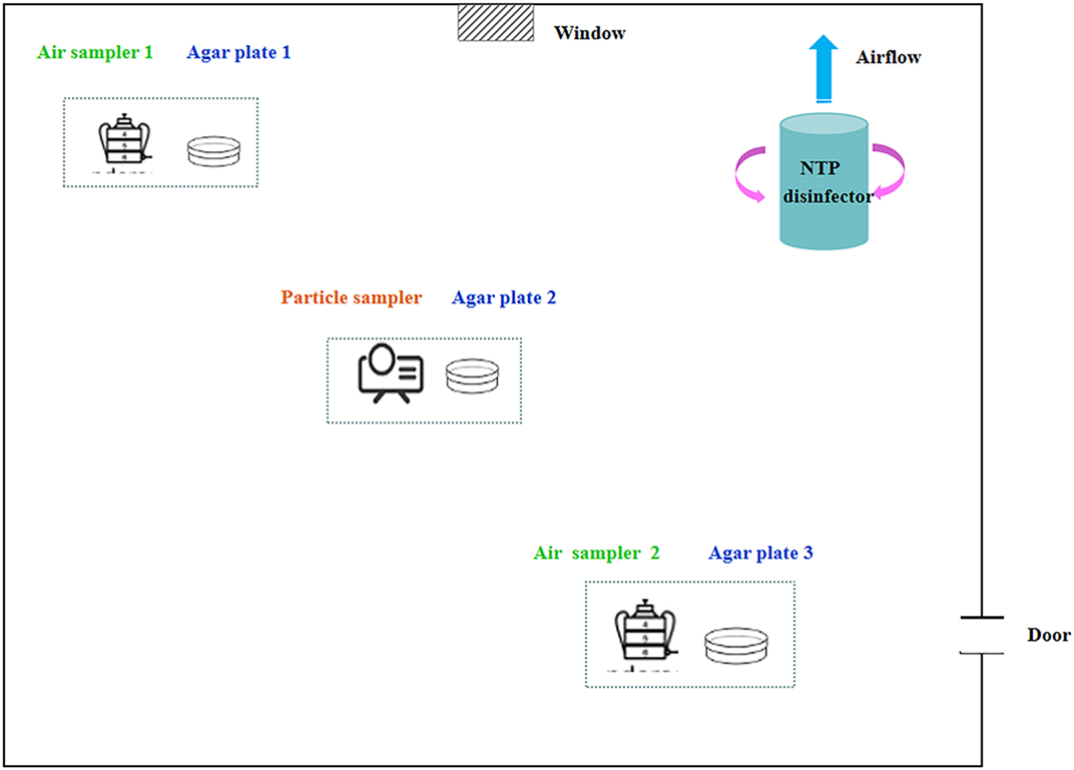
Location of the NTP disinfector and sampling points for airborne bacteria and PM in the Pulmonary Function Clinic.

Planktonic bacterial concentrations were determined using two six-stage Andersen microbial air samplers placed near the window and door (1.2 m height). Sampling was conducted for 5 min at an airflow rate of 28.3 L/min at 0, 30, 60, 90, and 120 min of continuous operation of the NTP air disinfector. Subsequently, the samplers were disinfected as described earlier. Sedimentary bacteria were assessed using three 90-mm nutrient agar plates placed diagonally at 1.2 m above the floor and exposed for 5 min, and incubated for 37 °C for 48 h. The PM_10_ concentrations were calculated using a particle sampler (8530, TSI, Inc., USA) placed centrally at 1.2 m above the floor (Fig. 1).

Two observers recorded temperature, relative humidity, number of indoor occupants, and personnel flow. The number of indoor occupants was defined as the number of individuals present in the room during sampling, while personnel flow indicated the total number of individuals entering and leaving the clinic between sampling points. The experiments were repeated on six different days.

### Statistical analysis

Planktonic bacterial counts were log-transformed prior to analysis. Continuous variables are presented as mean ± standard deviation or median and interquartile range (IQR). Group comparisons were performed using Student’s t-test, one-way analysis of variance (ANOVA), or Wilcoxon rank sum test. Statistical analyses were conducted using the R software (version 4.4.0; R Project for Statistical Computing), with a two-tailed *P* < 0.05 indicating statistically significant difference.

## Results

### Effectiveness of PM removal

**Fig. 2:**
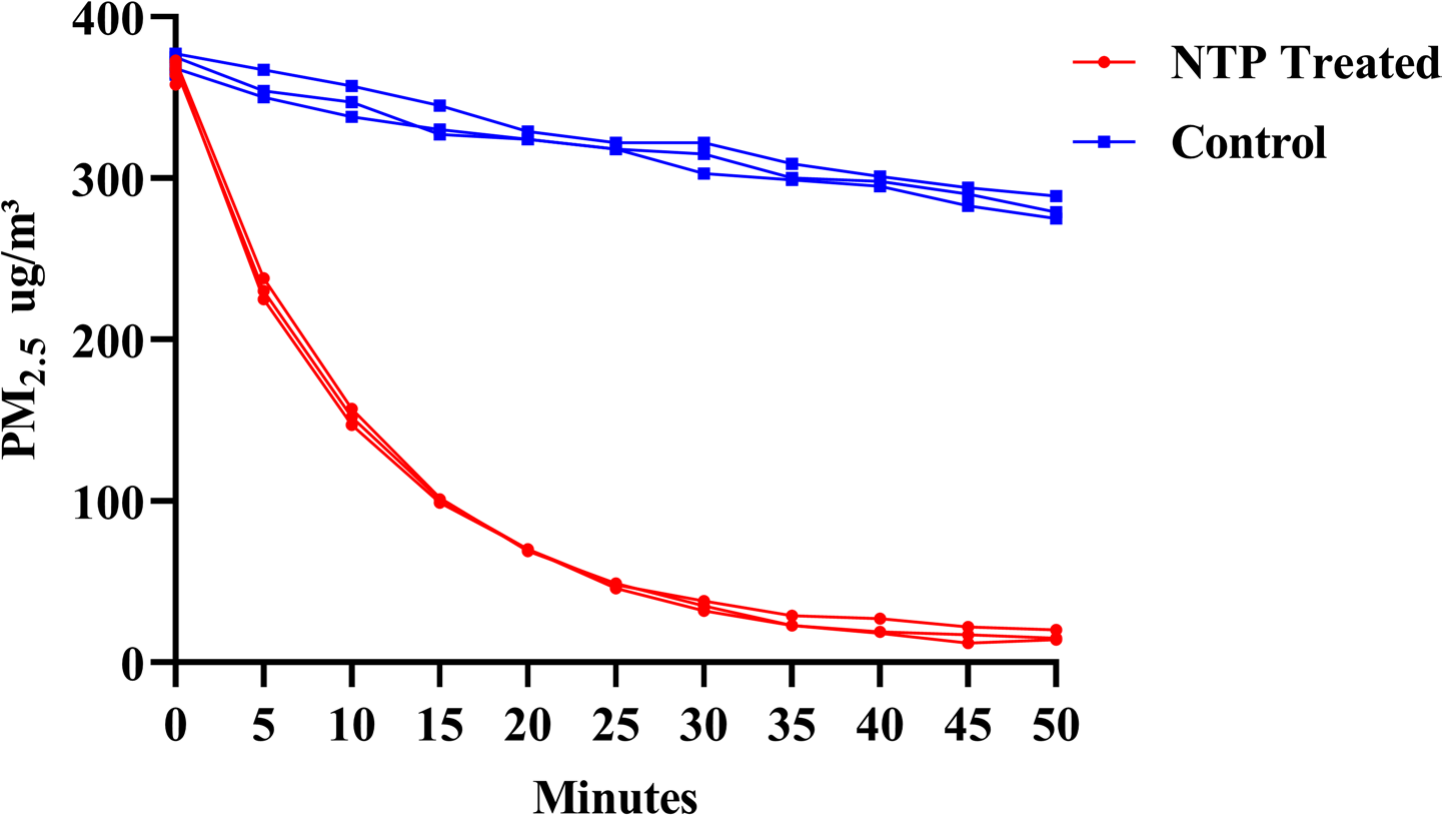
Removal efficiency of NTP for PM in the chamber at different treatment times.

The initial PM_2.5_ concentration was 367.11 ± 4.62 and 371.44 ± 4.98 µg/m^3^ in the NTP and control groups, respectively. At the end of the experiment (50 min), the PM_2.5_ concentration decreased to 16.33 ± 3.21 µg/m^3^ in the NTP group, whereas it remained at 281.00 ± 7.21 µg/m^3^ in the control group (*P* < 0.01). After 50 min, the decline in PM_2.5_ concentration in the NTP group reached a plateau, with no further substantial reduction. The natural decay rate of PM_2.5_ at each time point was calculated as follows: 3.9% (5 min), 6.5% (10 min), 10.1% (15 min), 12.3% (20 min), 14.0% (25 min), 15.6% (30 min), 18.5% (35 min), 19.8% (40 min), 22.2% (45 min), and 24.3% (50 min). After adjusting for natural decay, the actual removal rates of the NTP group at each time points were 34.53%, 55.72%, 69.50%, 78.36%, 84.90%, 88.70%, 91.64%, 92.76%, 94.05%, and 94.12%. The PM_2.5_ concentrations decreased by > 50% within the initial 10 min, reached approximately 85% reduction at 25 min, and subsequently exhibited minimal decline (Fig. 2).

### NTP disinfection of indoor air under unoccupied condition

Field tests were conducted in a classroom on four different days (two days for NTP disinfection and two days for control). To verify the disinfection efficacy of NTP, sampling was performed immediately after occupants had left to ensure sufficient baseline bacterial concentrations. The temperature was 24.96 °C ± 0.34 °C and relative humidity was 70.47% ± 2.21%. The baseline (0 min) airborne bacterial concentrations did not significantly differ between the groups (2.99 ± 0.15 log_10_ CFU/m^3^ in the NTP group vs. 3.06 ± 0.04 log_10_ CFU/m^3^ in the control group; *P* = 0.279).

**Fig. 3:**
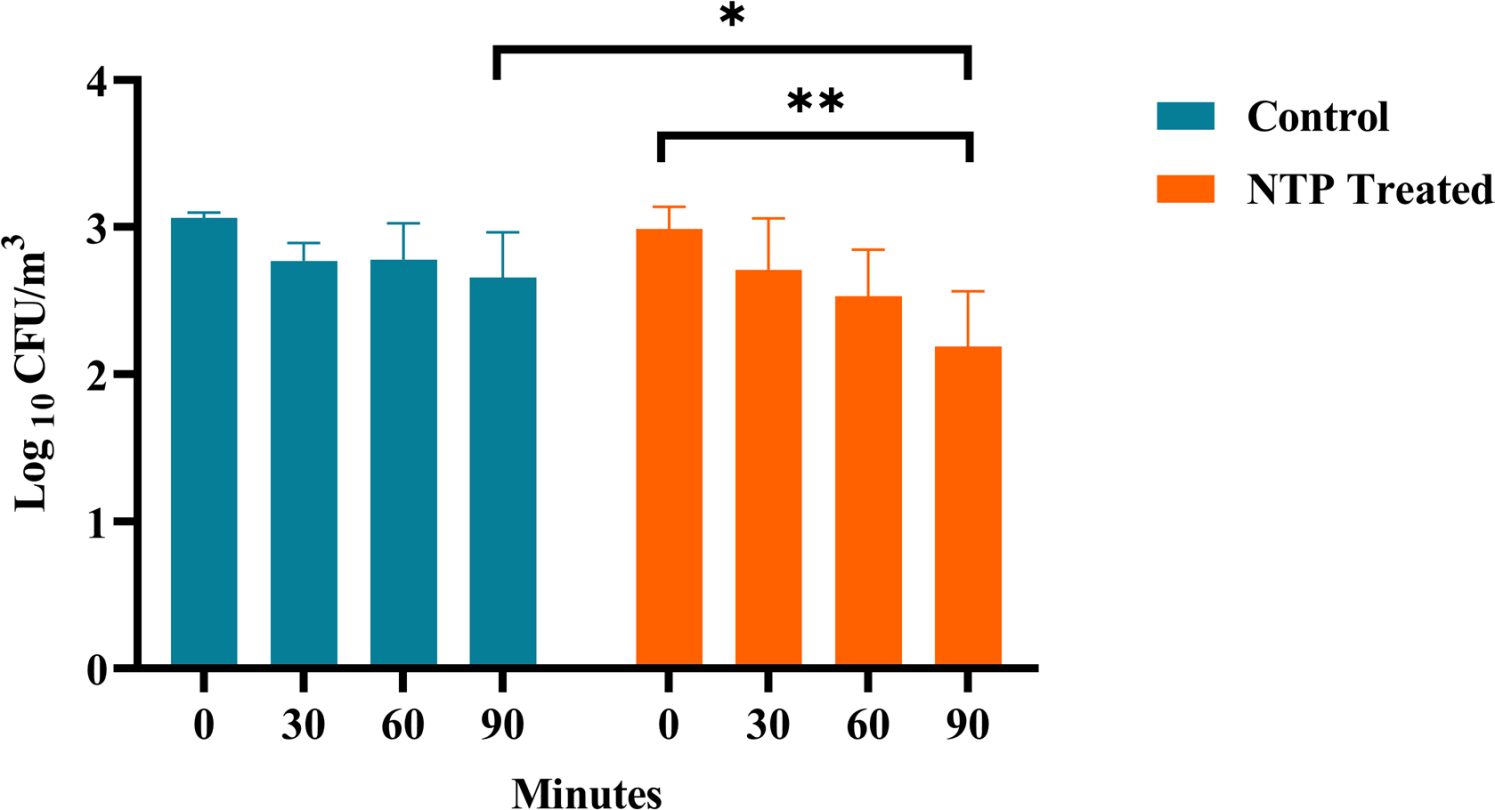
Effects of NTP disinfection at different time points. (*P < 0.05, **P < 0.01).

**Fig. 4:**
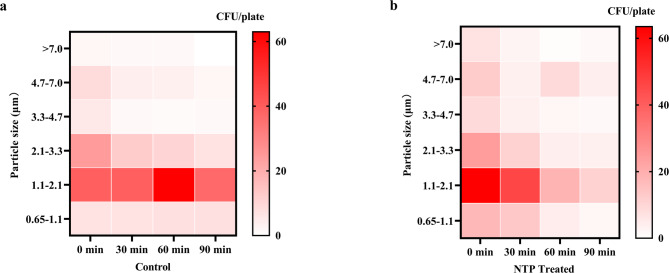
Bacterial counts across particle-size fractions in indoor air for the control group (a) and NTP group (b).

At 90 min (end of the test), the mean airborne bacterial concentration was significantly reduced in the NTP group, when compared with that in the control group (2.19 ± 0.38 vs. 2.66 ± 0.31 log_10_ CFU/m^3^; *P* = 0.039). Besides, in the NTP group, the airborne bacterial concentrations at 60 (2.53 ± 0.32 log_10_ CFU/m^3^) and 90 min were significantly lower than that at 0 min (*P =* 0.01 and *P <* 0.01, respectively) (Fig. 3). Particle-size distribution analysis showed the highest bacterial load (CFU/plate) in the 1.1–2.1-µm fraction, which progressively declined with increasing disinfection duration (Fig. 4).

### NTP disinfection of indoor air under real-world (occupied) conditions

**Fig. 5:**
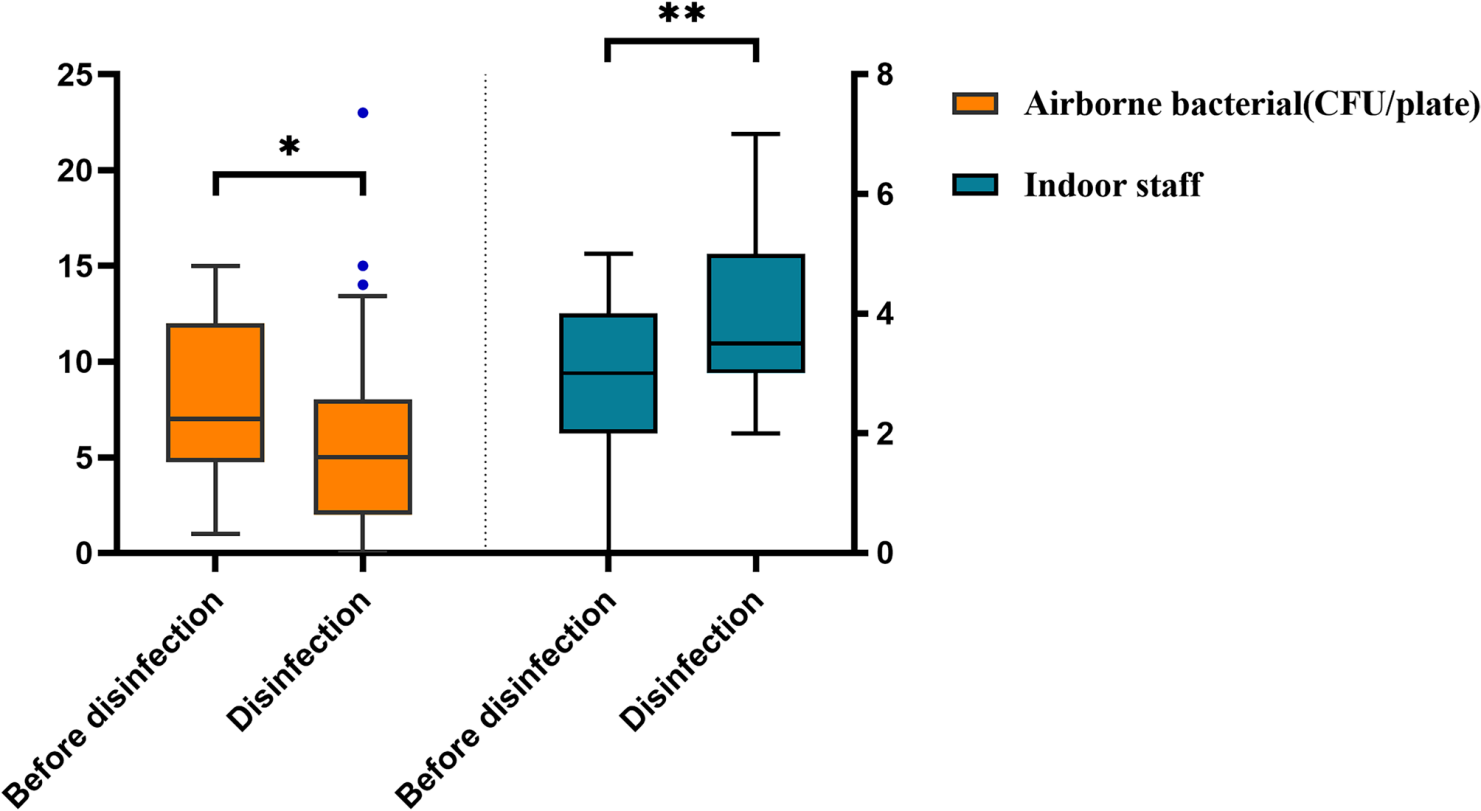
Sedimentary bacterial counts and indoor occupancy before and during NTP treatment.

The environmental and human-related variables are summarized in Table 1. A total of 90 samples of sedimentary bacteria were analyzed. The median bacterial counts were 7.00 CFU/plate (IQR: 5.00–11.80) before disinfection (0 min) and 5.00 CFU/plate (IQR: 2.00–7.50) during the disinfection process (*P* = 0.029). The mean number of indoor occupants increased from 3.00 (IQR: 2.00–4.00) before disinfection to 3.50 (IQR: 3.00–5.00) during the disinfection process (*P =* 0.028) (Fig. 5). The bacterial and PM concentrations at different time points are shown in Table 2.

**Table 1:** Environmental and human-related factors in field tests.

Items	Before disinfection	Disinfection
	Mean/Median (IQR)	Mean/Median (IQR)
Temperature (°C)	23.00 (22.75–24.00)	21.00 (20.00–23.00)
Relative humidity (%)	65.17 ± 15.90	73.17 ± 9.30
Number of indoor staff	3.00 (1.50–4.25)	3.50 (3.00–5.00)
Number of personnel flows	21.83 ± 8.66	23.88 ± 8.17

**Table 2:** Concentrations of airborne bacteria and PM at different disinfection time points.

Times	Median number of Indoor staff	Sedimentary bacteria (CFU/plate)	Planktonic bacterial (log_10_CFU/m^3^)	PM_10_ (ug/m^3^)
0	3.00	7.00 (IQR: 4.75–12.00)	2.91 ± 0.18	29.00 (IQR: 12.75–93.25)
30	3.00	3.00 (IQR: 2.00–12.00)	2.85 ± 0.22	18.50 (IQR: 7.75–26.25)
60	3.50	5.50 (IQR: 2.00–8.50)	2.94 ± 0.22	13.50 (IQR: 7.75–26.25)
90	3.00	3.00 (IQR: 2.00–8.00)	2.81 ± 0.28	13.00 (IQR: 5.25–19.25)
120	4.50	5.50 (IQR: 2.00–8.25)	2.84 ± 0.26	20.00 (IQR: 8.50–25.00)

**Fig. 6:**
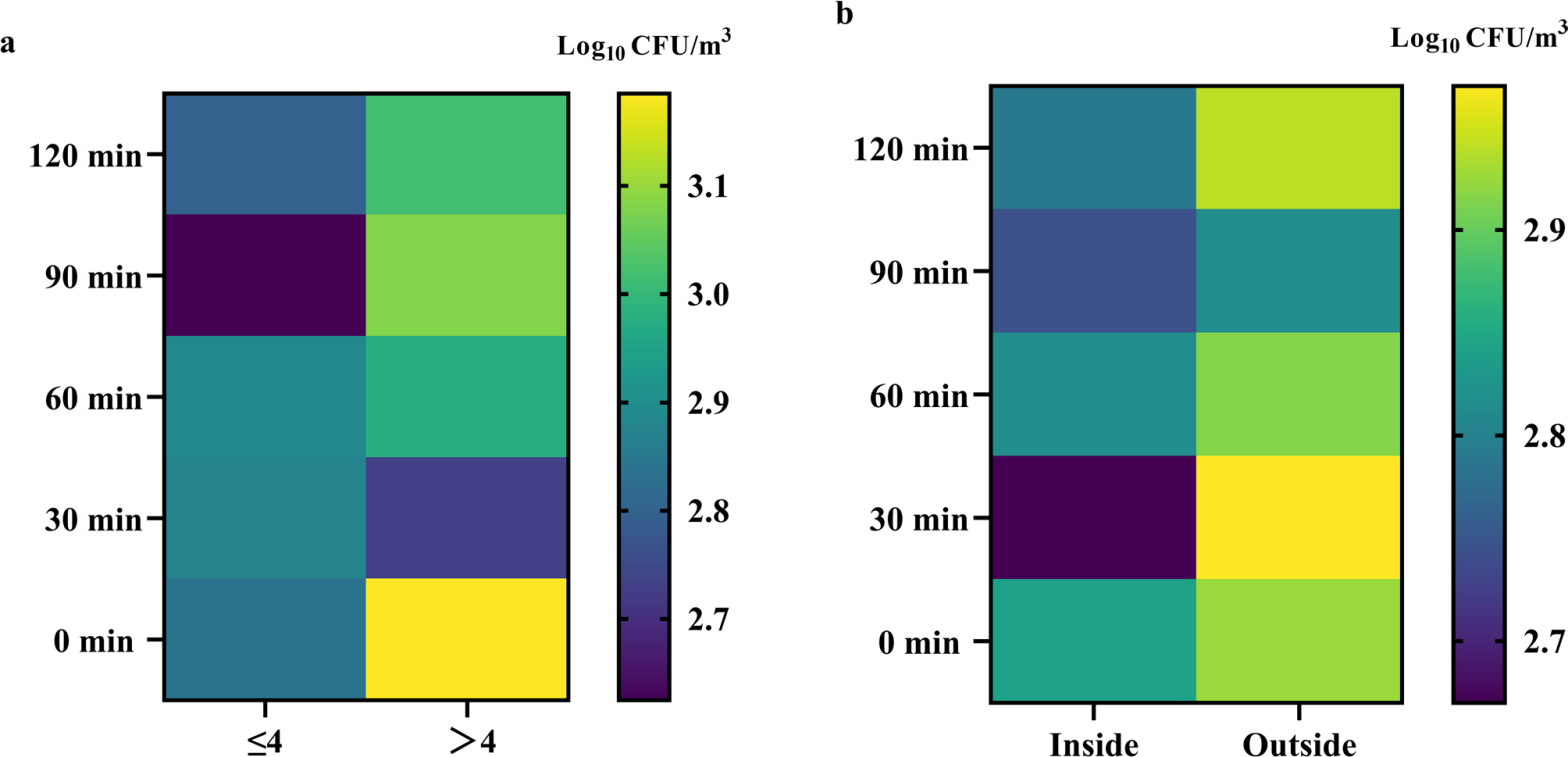
Planktonic bacterial concentrations associated with different indoor occupancy (a) and sampling locations (b).

A total of 60 samples of planktonic bacteria were analyzed. The mean airborne bacterial concentrations were 2.91 ± 0.18 log_10_ CFU/m^3^ before disinfection (0 min) and 2.86 ± 0.24 log_10_ CFU/m^3^ during the disinfection process; however, this difference was not statistically significant (Table 2). Human activities significantly influenced airborne bacterial levels, with concentrations higher at door-adjacent sampling points than those at window-adjacent locations (2.94 ± 0.18 vs. 2.80 ± 0.26 log_10_ CFU/m^3^; *P =* 0.016). Besides, the airborne bacterial concentrations were higher when the number of indoor occupants was ≥ 4, when compared with those when the number of indoor occupants was < 4, at 3.02 log_10_ CFU/m^3^ (IQR: 2.83–3.14) vs. 2.85 log_10_ CFU/m^3^ (IQR: 2.74–2.96), respectively (*P =* 0.033) (Fig. 6).

According to EU residential air guidelines^27^ and standards for indoor airborne microorganisms established by the Chinese Academy of Sciences^28^, 1000 CFU/m^3^ is considered as the upper acceptable airborne bacterial limit. In this study, planktonic bacterial concentrations in 16 samples (26.7%) were > 1000 CFU/m^3^, with average number of indoor occupants being 5.00 (IQR: 3.00–5.00). In contrast, in samples with bacterial concentrations < 1000 CFU/m^3^, the average number of occupants was lower [3.00 (IQR: 3.00–4.00)], representing a decrease of approximately two people (*P* = 0.028). Furthermore, consistent with previous findings, the highest bacterial load was observed in the 1.1–2.1-µm fraction, and the PM_10_ concentrations during the experimental period are presented in Table 2.

## Discussion

This study evaluated the removal and disinfection efficiency of NTP for PM and airborne bacteria, with particular focus on its performance under real‑world settings. In the chamber experiments, NTP demonstrated rapid and substantial PM_2.5_ removal. After adjustment for natural decay, the PM_2.5_ concentrations decreased by approximately 55% within 10 min of NTP treatment and by about 90% after 30 min of NTP treatment. In field settings, the PM_10_ levels also declined following NTP operation; however, the magnitude of reduction was not significant, possibly owing to continuous human activities. The ability of NTP to remove PM is mainly attributed to oxidation processes. Within the plasma zone, PM undergoes oxidation, aggregation, and fragmentation under the action of reactive species (e.g., O_3_ and NO_2_). Some particles undergo surface oxidation, resulting in disruption of the graphitic microstructure into a more disordered state. Larger soot agglomerates are oxidatively fragmented into smaller, less stable fragments that are more readily oxidized and subsequently eliminated, thereby leading to reductions in both particle mass and number concentrations^29,30^.

Similarly, previous studies on PM removal by plasma have also reported consistent findings. For instance, Ji et al.^20^ proved that NTP modified the microstructure and oxidation characteristics of PM released from diesel engines, supporting the use of NTP as an efficient post-treatment technology for diesel exhaust. Yang et al.^31^ observed reduction in relative particle mass and altered particle size distribution in commercial charbroiling emissions following plasma treatment. Hernández-Díaz et al.^32^ reported approximately 50% reduction in maximum particle concentration using NTP treatment, when compared with the control. In the field experiment, the PM concentration was 29 ug/m^3^ before disinfection and decreased to a minimum of 13 ug/m^3^ during disinfection. Compared with filtration methods integrated into centralized HVAC (Heating, Ventilation and Air Conditioning) systems, which have reported PM removal efficiencies of > 80% in classrooms^33^, the PM reduction observed in this study was not significant. Several factors may explain this finding. First, the effective sample size for PM measurements in this study was limited; only one sampling point was used, corresponding to a theoretical sample size of 30, which may not have been sufficient to show a statistically significant difference. Second, the air disinfector used in this study operated via indoor air recirculation, and its circulating airflow rate was substantially lower than that of centralized HVAC systems, resulting in smaller treated air volume under comparable conditions. Further studies with improved experimental design are therefore needed to more accurately determine the effectiveness of NTP for indoor PM removal.

NTP has attracted increasing attention as a promising air disinfection technology. Laboratory studies have demonstrated that NTP exhibits high inactivation efficiency against various types of bioaerosols. It has been reported that the single-pass efficiency of NTP for *E. coli* or *Staphylococcus lentus* aerosols was > 99%, with residence time of about 3.6 ms^34,35^. Bisag et al.^17^ achieved a 3.7-log reduction in bacterial bioaerosols and complete elimination of SARS-CoV-2 RNA with a residence time of < 0.2 s in the plasma zone. In these studies, bioaerosols were passed once through the plasma region and their concentrations were measured upstream and downstream of the plasma zone. In the present study, the estimated residence time was approximately 0.7 s based on the circulating airflow rate and the volume of the air disinfector. In addition, zone-disinfection studies have also confirmed the effectiveness of NTP in reducing sedimentary and planktonic bacteria in enclosed chambers^18^. Baek et al.^16^ showed complete zone disinfection effect of NTP on airborne *E. coli*, *Staphylococcus epidermidis*, and *M. smegmatis* in a 60-L chamber, while our previous study confirmed effective inactivation of airborne *Staphylococcus albus* and Φ174 bacteriophage by NTP in a 20-m^3^ chamber^22^. However, these investigations were conducted under strictly controlled laboratory conditions, whereas real-world environments involve more complex bioaerosols and variable environmental factors that are difficult to regulate, thereby making field validation essential.

This study implemented field experiments under both unoccupied and occupied conditions. In the classroom setting, when compared with the control group, 90 min of NTP treatment significantly decreased airborne bacterial concentrations from about 1000 to 200 CFU/m^3^, corresponding to a decrease of nearly 80%. Cheng et al.^25^ reported that the use of a plasma circulation air sterilizer in computed tomography rooms during the COVID-19 pandemic reduced airborne bacterial concentrations by about 83% (from 120 CFU/m^3^ to 20 CFU/m^3^) after 60 min of disinfection. In comparison, our results showed a 65% reduction of airborne bacterial concentrations after 60 min of disinfection. This difference may be attributed to the variations in circulating airflow rates between the devices used in the two studies (450 m^3^/h vs. ≥1000 m^3^/h). Similarly, Wang et al.^26^ compared pulsed xenon UV devices with an NTP air disinfector in blood sampling rooms and observed decreases in sedimentary bacteria (from 1 CFU/cm^2^ to 0 CFU/cm^2^) following both the interventions.

In the present study, particle size distribution analysis revealed that the highest bacterial concentrations occurred in the 1.1–2.1-µm fraction. Similar conclusions have also been reported in other related studies. For instance, it has been reported that the total airborne microorganisms exhibited a distribution pattern, peaking at 1.1–2.1 μm^37,38^, and that the viability of bacteria is higher in fine particles (< 2.1 μm) than in coarse particles^36^. As bioaerosols within the size range of 1.1–2.1 μm can penetrate into the terminal bronchi^37^, reducing fine PM to relatively low levels is essential for the prevention of PM-related diseases.

In the Pulmonary Function Clinic room test, routine clinical activities were continued during NTP operation, and sedimentary bacteria, planktonic bacteria, and PM were simultaneously monitored to comprehensively evaluate the disinfection and purification efficacy of NTP under real-world conditions. Although all the three indicators decreased after NTP treatment, when compared with no treatment, the reduction was insignificant, which may be attributed to human activities. Notably, the number of indoor occupants during NTP operation was higher than that before disinfection. Despite increased human activity, the airborne bacterial concentrations and PM levels did not increase during NTP treatment, supporting the effectiveness of NTP under practical conditions.

This study further demonstrated that human activities significantly influence indoor bacterial concentrations. Higher bacterial levels were observed when the number of indoor occupants exceeded 4 or when sampling was conducted near the door. Consistent with this observation, Yang et al.^38^ reported higher concentrations of bacteria and fungi at the entrance of an intensive care unit, when compared with those at the end of the ward, highlighting that frequent human movement increases indoor air contamination.

The present study design is comparable to investigations of air contamination in operating rooms (ORs), where continuous air purification is maintained during surgical procedures. Studies in OR environments have indicated that human activities and door openings increase the airborne bacterial load during surgery^39,40^. Multiple regression analysis by Fu et al.^41^ showed that an increase in the number of indoor occupants in OR was associated with an increase of 4.93 CFU/m^3^ (95% CI: 1.47–8.38) in airborne bacterial load. Stauning et al.^42^ reported that each door opening corresponded to a 0.2% increase in CFU/m^3^, and each additional person in the OR contributed to a 2.5% increase in airborne CFU/m^3^. These findings suggest that the number of patients and staff in clinic rooms should be restricted and unnecessary door openings should be minimized.

Overall, this study comprehensively evaluated the removal efficacy of NTP against airborne bacteria and PM through both laboratory and field investigations, providing a scientific basis for the practical application of NTP in air disinfection. However, several limitations should be acknowledged. Given that the present study was a short-term field trial, the effects of climatic variables such as temperature and relative humidity on NTP performance could not be comprehensively assessed. In addition, fungal aerosols and bacterial community diversity were not analyzed, limiting insights into broader microbiological dynamics. As this study represents an ongoing research program advancing from laboratory experiments to field trials, these aspects are currently under investigation and will be addressed in a phased and systematic manner in future works.

## Conclusion

This study systemically evaluated the efficacy of NTP for air disinfection and PM purification. Laboratory experiments demonstrated that NTP rapidly eliminated airborne PM, achieving approximately 90% reduction in PM after 30 min of treatment. In unoccupied field settings, NTP disinfection effectively reduced airborne bacterial contamination. In occupied field trials, despite interference from human activities, NTP still reduced the levels of airborne bacteria and PM. Bacteria in the 1.1–2.1-µm size range represented the dominant fraction of airborne contamination. These findings suggest that the number of individuals present in the clinic rooms and the frequency of door openings should be appropriately controlled. Overall, NTP represents a promising air disinfection technique for improving indoor air quality and preventing airborne diseases in public environments.

## Data Availability

The data presented in this study are available on request from the corresponding author.
